# Antifungal activity of Myriocin on clinically relevant *Aspergillus fumigatus* strains producing biofilm

**DOI:** 10.1186/s12866-015-0588-0

**Published:** 2015-10-30

**Authors:** Federica Perdoni, Paola Signorelli, Daniela Cirasola, Anna Caretti, Valentina Galimberti, Marco Biggiogera, Paolo Gasco, Claudia Musicanti, Giulia Morace, Elisa Borghi

**Affiliations:** Department of Health Sciences, Università degli Studi di Milano, Polo Universitario San Paolo, Blocco C, ottavo piano, via di Rudinì 8, 20142 Milan, Italy; Department of Biology and Biotechnology, Università degli Studi di Pavia, Pavia, Italy; Nanovector Srl, Torino, Italy

**Keywords:** Fungal infections, Ceramide, Nanocarriers

## Abstract

**Background:**

The human pathogenic mold *Aspergillus fumigatus* is able to form a complex biofilm embedded in extracellular matrix. Biofilms confer antimicrobial resistance and it is well known that aspergillosis is often refractory to the conventional antifungal therapy. The treatment of biofilm-related infections poses a significant clinical challenge on a daily basis, promoting the search for new therapeutic agents.

Our aim was to exploit the modulation of sphingolipid mediators as new therapeutic target to overcome antifungal resistance in biofilm-related infections.

**Results:**

Antifungal susceptibility testing was performed on 20 clinical isolates of *Aspergillus fumigatus* and one reference strain (*A. fumigatus* Af293) according the EUCAST protocol. Sessile MICs were assessed on 24-h preformed-biofilm by means of XTT-reduction assay. Myriocin (0.25–64 mg/L), a commercial sphingolipid synthesis inhibitor, was used. The MEC_50_ value (mg/L) of Myriocin was 8 (range 4–16) for both planktonic and sessile cells.

Drug-induced morphological alterations were analyzed by optical and electron microscopy (TEM) on 24h preformed *A. fumigatus* Af293 biofilms.

An evident hyphal damage, resulting in short, stubby, and highly branched hyphae was observed by optical microscopy. At 24h, TEM studies showed important morphological alterations, such as invaginations of the cell membrane, modification in the vacuolar system and presence of multilamellar bodies, in some cases within vacuoles.

**Conclusions:**

The direct antifungal activity, observed on both planktonic and sessile fungi, suggests that inhibition of sphingolipid synthesis could represent a new target to fight biofilm-related *A. fumigatus* resistance.

## Background

The ubiquitous environmental mold *Aspergillus fumigatus* is one of the most common fungal pathogens. The risk of developing fungal infections is increasing in patients with underlying debilitating diseases, such as cancer, chronic lung diseases, transplantation or immune system impairment [1, 2].

*A. fumigatus* can cause a variety of diseases, ranging from invasive pulmonary aspergillosis and aspergilloma to allergic syndromes such as allergic rhinitis and asthma, and allergic bronchopulmonary aspergillosis (ABPA) [3]. In chronic forms of aspergillosis *A. fumigatus* has been demonstrated to develop a thick biofilm that promotes its persistence [3, 4].

Bacteria and fungi are able to secrete polymers that function as scaffold for biofilm formation. These slime-embedded microbial communities endow the pathogens with an intrinsic ability to escape immune-surveillance, and are also poorly or null penetrable by most of the antimicrobial drugs [5].

Antifungal resistance is emerging in *Aspergillus* species, and it is becoming a major health problem in biofilm-related infections. Failure of antifungal treatment can be due to host factors, to the pharmacokinetic and pharmacodynamics parameters of the drug or to morphological, reproductive modalities and biofilm production of the fungus itself [6, 7].

Due to the common eukaryotic structure of fungi and humans, a limited number of antifungal drugs is tolerated by the host and available for therapeutic purposes, and new targets as well as innovative strategies to overcome primary and biofilm-related resistance are needed.

Several sphingolipid metabolism inhibitors have been demonstrated to exert a broad-spectrum antifungal activity [8]. Sphingolipids (SPLs) are a class of molecules with structural and signaling activities conserved from fungi to humans. Many studies have demonstrated that sphingolipid mediators or modulators are involved in infection-related mechanisms [9–11]. Ceramides are the central molecules in SPLs and glycosphingolipids (GSLs) biosynthesis. The biosynthesis of sphingolipids starts with the condensation of a fatty acyl CoA, usually palmitoyl CoA, with serine, which is catalyzed by serine palmitoyl transferase (SPT), a common enzyme in the biosynthetic pathway of SLPs of both fungal and human cells and a possible common target of sphingolipid metabolism inhibitors.

Myriocin is a specific inhibitor of de novo sphingolipid synthesis and its administration to human cells causes a re-arrangement of the endogenous pools of sphingolipids and an overall inhibition of proliferation without triggering cell death. Myriocin was used in research studies to inhibit highly proliferative cancerous cells with no sign of toxicity for normal cells [12, 13]. Moreover its effect as an immunomodulator/immunosuppressant in vivo has been reported but there is no clear evidence of specific mechanism underlying this activity of the compound [14].

Nanocarriers lung delivery of Myriocin, a commercial inhibitor of SPT catalytic activity, has been recently shown to efficiently reduce the inflammatory milieu in cystic fibrosis mice, and consequently to reduce the persistence of *Pseudomonas aeruginosa* infection [15].

In consideration of the opportunistic nature of fungal infection that takes advantage of patient’s pre-existing inflammatory conditions, and to the strict dependency of fungal survival on SPL synthesis, our aim was to exploit the Myriocin antifungal activity to overcome biofilm-related fungal resistance. We here demonstrate that solid lipid nanocarriers-mediated Myriocin delivery represents a promising tool in biofilm-related aspergillosis.

## Results

### Myriocin in vitro susceptibility testing and time-killing assay

To assess the direct antifungal activity of Myriocin, we performed the standard antifungal susceptibility testing on *A. fumigatus* conidia.

**Fig. 1:**
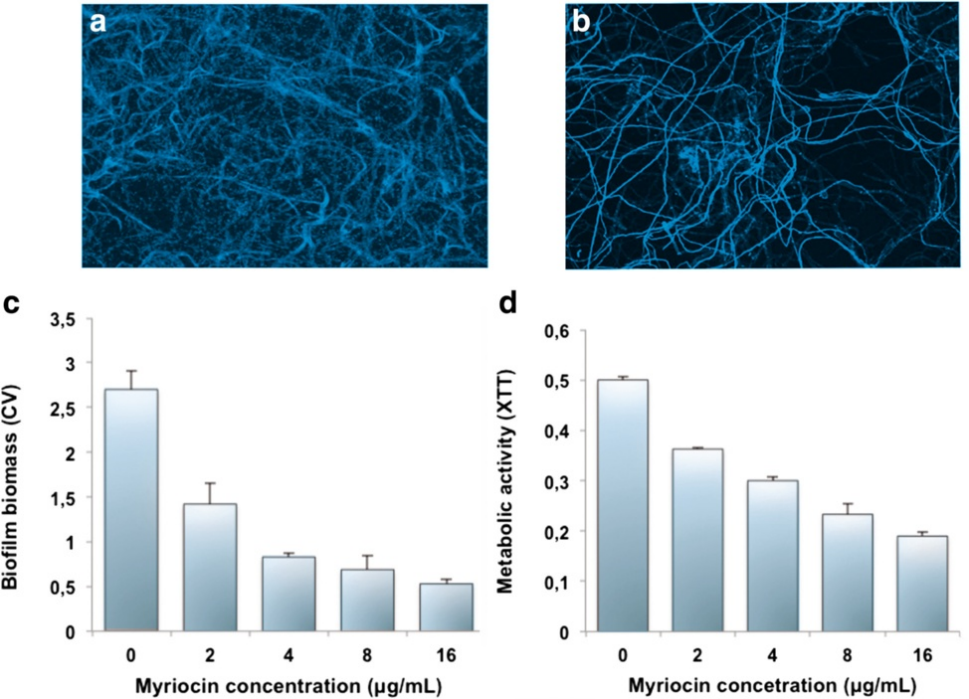
Effect of Myriocin on *A. fumigatus* biofilms. Biofilms exposed to Myriocin were characterized by CLSM (20X): (**a**) untreated biofilm, and (**b**) biofilm 24h post-Myriocin (4 mg/L). The drug efficacy was also assessed by measuring the fungal biomass (**c**), and the biofilm metabolic activity (**d**)

The minimum effective concentration of Myriocin inducing morphological alteration of *A. fumigatus* hyphae (MEC_50_) was 8 mg/L (range 4–16). The drug-induced alterations observed microscopically are shown in Fig. 1b, and compared to the untreated fungus (panel a).

**Fig. 2:**
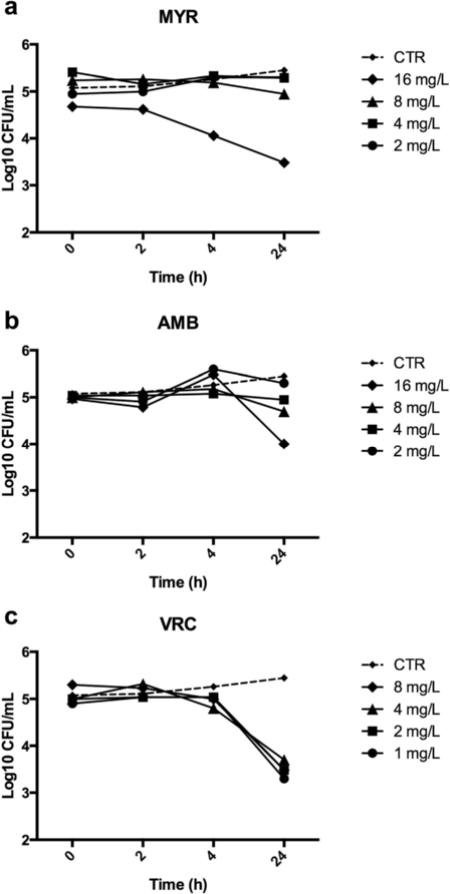
Time-kill curves of Myriocin against *Aspergillus fumigatus* Af293. To assess the possible fungicidal activity of Myriocin, the reference strain Af293 was exposed to various concentrations of Myriocin, from 2 to 16 mg/L (**a**). For comparative purpose, two conventional antifungal drugs, amphotericin B (AMB, **b**) and voriconazole (VRC, **c**) were tested

The time-kill curves performed on the reference strain Af293 demonstrated that within the 24 h of Myriocin exposure, there was only a limited concentration-dependent killing. In fact, the difference to the control was about 2 log10 CFU/mL for the higher Myriocin concentration (Fig. 2).

We then evaluated the antifungal activity of Myriocin on 24 h preformed *A. fumigatus* biofilms. The sessile MIC_50_, measured by means of XTT-reduction assay after 24 h treatment, was 8 (range 4–16 mg/L).

We also observed a dose-dependent progressive reduction of both biomass and metabolic activity of the biofilms, as determined by CV and XTT assays respectively, in response to different Myriocin concentrations. Figure 1c, d reports the significant (*p* < 0.05) reduction of both parameters.

### Effect of Myriocin treatment on fungal preformed biofilms morphology and structure

To investigate the morphological and structural changes induced by Myriocin on preformed fungal biofilms, *A. fumigatus* Af293 was used for CLSM and TEM microscopic evaluation after 24 h following exposure to 4 mg/L Myriocin.

Biofilm thickness was measured by CLSM z-sectioning. The inhibition of sphingolipid de novo synthesis highly influenced the production of biofilm biomass in *A. fumigatus* Af293, with an overall reduction of almost 60 % (32 μm ± 1.61 versus 13.4 ± 0.96 μm, *P* < 0.0001).

**Fig. 3:**
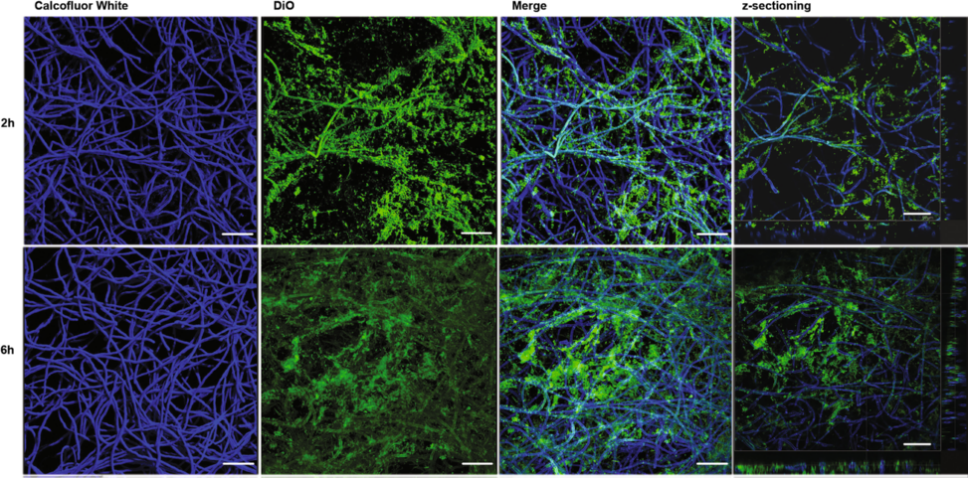
Confocal laser scanning microscopy images of two-day-old biofilms of *A. fumigatus* strain Af293. The fungal biofilms were stained with calcofluor white (blue staining). SLNs labelled with DiO (green staining) were then added and the penetrance measured after 2 h (upper panels) and 6 h (lower panels). The rectangular micrographs on the sides (right panels) represent the x–z plane and y–z optical cross sections through the thickness of the biofilms. The images shown (CLSM 20X) are representative of three independent experiments. Bar = 50 μm

In order to develop a strategy for biofilm effective drug delivery, the use of SLNs as Myriocin carrier was evaluated by means of labeled particles. We demonstrated that SLNs are able to penetrate the fungal biofilm (Fig. 3), and to reach the bottom layer of the biofilm within 6 h.

Ultrastructural imaging was performed by TEM analyses on *A. fumigatus* Af293 biofilms, treated or not with Myriocin, at various time points (4, 24 and 48h).

**Fig. 4:**
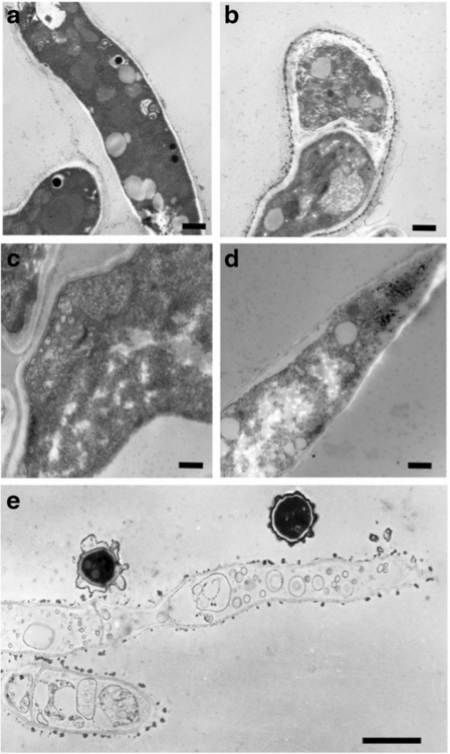
Ultrastructural imaging of *A. fumigatus* Af293 biofilm morphology treated or not with Myriocin (4 mg/L). **a** 48h untreated biofilm: normal fungal cells morphology (12000X original magnification -o.m.; Bar = 600 nm); **b** 4h post-Myriocin: the fungal cell showed some alterations of the inner membrane that became non homogeneous (12000X o.m.; Bar = 600 nm); **c** 24h post-Myriocin: the plasma membrane showed several disorganized invaginations associated with cell wall detachment (20000X o.m.; Bar = 400 nm); **d** 24h post-Myriocin: the hyphae showed a cell wall collapsed, and a degenerated cytoplasm highly vacuolarized; none of the cell organelles was recognizable. (12000X o.m.; Bar = 600 nm); **e** 48h post-Myriocin: a large part of the fungal cells is damaged and disrupted (12000X o.m.; Bar = 2.5 μm)

The ultrastructural imaging of *A. fumigatus* biofilms treated with Myriocin (4 mg/L) showed that there was a progressive loss of cell structure both at cell membrane and at cytoplasmic level (Fig. 4). 4 h after treatment, the fungal cells already showed some alterations of the inner membrane that became irregular in thickness and density, while internal organelles like mitochondria were well preserved (Fig. 4b). After 24 h of Myriocin treatment, many cells were damaged, showing cell wall detachment from plasma membrane, invaginations of the plasma membrane, presence of multilamellar bodies, and reduced density of cytosolic matrix. The internal structure was less definite and showed higher density (Fig. 4c). In some cases, the hyphae showed a cell wall collapsed (Fig. 4d). Cytoplasm was clearly degenerated due to high vacuolization and no organelle was still recognizable. When Myriocin treatment was prolonged up to 48 h, a large part of the fungal structure was damaged and disrupted, and many cells looked empty (Fig. 4e).

### Effect of Myriocin treatment on fungal sphingolipid synthesis

**Fig. 5:**
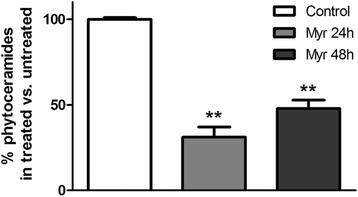
Phytoceramides content in *A. fumigatus* Af293 biofilms. 24h-preformed *A. fumigatus* biofilms were treated with Myriocin (4mg/L) for 24 and 48h. Bars represent ceramide levels in untreated biofilm (black bar) and in Myriocin-treated biofilm (stripes bar). Significance was evaluated by t-test versus control untreated biofilm (**, *p* < 0.01)

To assess the direct activity of Myriocin on sphingolipid de novo synthesis in fungi, we measured intracellular phytoceramides content, a key intermediate, of *A. fumigatus* biofilms in presence or absence of the drug. Phytoceramide is a key sphingolipid intermediate, maintained in a homeostasis concentration in normal condition or increased and actively signaling upon a variety of stresses. As expected, we observed a significant decrease (about 70 %) of phytoceramides level in *A. fumigatus* biofilm exposed to Myriocin (Fig. 5)*.*

## Discussion

Our study shows that Myriocin exerts a strong antifungal activity against *A. fumigatus*, by inhibiting conidia germination, and significantly reduces biofilm metabolism and biomass.

By optical microscope, we were able to demonstrate that Myriocin led to the presence of aberrant hyphal structures in *A. fumigatus* conidia, with increased branching and reduction in apical hyphal growth. Polarized growth is essential for the morphology of filamentous fungi and atypical hyper-branched hyphae are seen in the presence of active antifungal compounds [16].

In addition, Myriocin exerted antifungal activity against preformed fungal biofilms by reducing their biomass and metabolic activity, as clearly shown by the ultrastructural studies (Fig. 4).

The treatment with the sphingolipid de novo synthesis inhibitor resulted in a significant disadvantage for *A. fumigatus* growth. Our hypothesis is that sphingolipid synthesis inhibition acts as a fungistatic agent by affecting the maintenance of hyphae polarization. The resulting aberrant hyphal growth destabilizes the biofilm, and impairs its maturation and three-dimensional organization.

Polarized hyphal extension strongly correlates with the presence of specific structures in the hyphal tip. This complex, a vesicle transit station, has a key role in the hyphal morphogenesis, containing cell wall synthesis enzymes, ribosomes, microtubules, and actin that are required for a polarized growth [17, 18]. Cheng co-workers suggested that lipid rafts, sphingolipid-rich plasma membrane domains, could be involved in the polarity apparatus. The inhibition of sphingolipid de novo biosynthesis disrupts the actin organization at the tip, and subsequently the normal hyphal growth [19]. The disruption of actin patches at the hyphal tip could also explain the aberrant multiple short hyphal branches. The lipid rafts are also responsible of the selective inclusion of plasma membrane-anchored proteins that participate in the establishment and maintenance of the polarized growth [20].

The aberrant multiple short hyphal branching observed in this study could be related to disruption of actin patches induced by Myriocin. Thus, it is conceivable, given that sphingolipids are major components of all eukaryotic membranes, that the sphingolipid synthesis inhibitor Myriocin modulates the balance between neo-synthesis and recycle of sphingolipids in membrane, affecting rafts activities and signaling.

Moreover, other studies demonstrated that ceramide synthesis promotes, while inhibition of ceramide synthesis down regulates, the activity of sterol regulatory element binding protein (SREBP) [21], responsible for hypoxia adaptation, virulence and biofilm formation in *A. fumigatus* [22, 23]*.* On-going studies in our laboratory are currently investigating the hypothesis that Myriocin could exert its powerful fungistatic activity by regulating sphingolipids and ergosterol formation in *A. fumigatus*. Being hypha development depending on massive production of membranes, the inhibitor of sphingolipid synthesis is highly effective in impairing fungal growth via hypha elongation. This observation poses Myriocin as a powerful fungistatic, abrogating the possibility to establish fungal biofilms and to enhance fungal sensitivity to fungicidal drugs. The fungistatic activity has also been confirmed by time-kill assay. Indeed, as for the conventional antifungal drugs voriconazole and amphotericin B, we observed a dose-dependent effect that did not reach the arbitrary criterion determining fungicidal activity (99.9 % or 3-log-unit decrease) [24].

Further studies, in vitro and in vivo, are needed to investigate the possible association of Myriocin with conventional antifungals.

A contrasting evidence suggested that myriocin can exert adverse effect on fungal infection treatment, due to an overall immune response suppression induced in the host that would eventually favor pathogen colonization [25]. Although myriocin immunomodulatory activity was proved [14, 25], it is important to point out that the dose of the compound, used by Melo and coworkers (0.05 mg/L) in the *Galleria mellonella* model of *Candida* infection, is about ten folds lower than the one necessary for antifungal activity, reported by us and by others on fungal growth [26, 27]. It is evident that at low dose, and systemically administered, the compound did not have the chance to act directly on fungal growth but it was probably sensed by hemolymph circulating hemocytes, reducing the larvae defense response without affecting *Candida* invasion. In line with this hypothesis, the same authors questioned the possibility of a delayed antifungal effect of myriocin, at a late phase of pathogens infection [25].

The morphological status of fungi plays an essential role in the pathogenesis of fungal infections and in the response to antifungal treatment. *A. fumigatus* has been shown to become increasingly resistant to antifungal agents throughout morphological differentiation. In particular, its ability to form a thick biofilm, characterized by matrix production, results in a multifactorial resistance phenomenon [28]. Our in vitro results confirm that Myriocin exerts a powerful action in disrupting preformed *A. fumigatus* biofilm, suggesting that targeting sphingolipid metabolism could offer a new powerful tool against biofilm forming infectious fungi.

With our data we basically propose myriocin as antimicrobial agent in view of the need of pathogens to reproduce themselves fast in the host and to establish their colonies. This high metabolism rate phase of the invasion is impaired by Myriocin. Further studies are required to assess the dose, time and mode of treatment to obtain maximal fungal toxicity with no side effect for the host.

Finally, the encapsulation of antimicrobial agents into solid lipid nanocarriers could be a potential strategy to eradicate biofilms [29]. Our nanoparticles, due to their lipophilic nature, ensure homogeneous delivery into the deep biofilm structure, therefore decreasing the minimal inhibition concentration (MIC) of the drug. Since nanocarriers are known to provide drug stabilization and long lasting release, ad hoc formulation can be envisaged in anti-fungal therapies. Solid lipid nanocarriers size cut off for optimal drug delivery depends on the mesh size of the biofilm matrix and it has been tested for a few bacteria [30] and fungi, including *Candida* [31]. Our data assess the advantages of using minimal size (30–50 nm) solid lipid nanocarriers in fungal biofilm eradication, exhibiting a fast spread of the drug into biofilm layers, minimal effective dose and homogeneous delivery of poorly soluble drugs. The use of SLN is recommended with highly lipophilic drugs that hardly dissolve into vehicle solutions. Nanovectors not only ensue solubility but also efficacious membrane interaction and drug upload. In our case, the use of SLN improved significantly drug introduction into deep biofilm layers.

## Conclusions

This study demonstrated a direct antifungal activity of Myriocin on *A. fumigatus* biofilm, suggesting that inhibition of sphingolipid metabolism could represent a new target to overcome biofilm-related fungal infections. Further studies in vitro and in vivo are required to fully define the mode of action of this compound, especially the downstream signaling pathway involved in the inhibition of hyphae germination.

Nanocarrier delivery of Myriocin was shown to represent a promising tool for drug delivery into the fungal biofilms, with broader implications for healthcare applications.

## Methods

### Myriocin stock solution preparation

Myriocin powder (Sigma Aldrich, Milan, Italy) was weighted and dissolved in dimethyl sulfoxide by warming up at 37 °C, to a final concentration of 1 mM. Solution was sterile filtered and stored at -80 °C until used. This stock solution was diluted in Roswell Park Memorial Institute (RPMI) 1640 broth supplemented with 2 % of glucose and buffered with morpholinepropanesulfonic acid sodium salt.

### Myriocin loaded-solid lipid nanoparticles (SLNs)

Myriocin-loaded nanocarriers (30–50 nm-diameter solid lipid nanoparticles, SLNs) were prepared by Nanovector srl (Turin, Italy), and a 1 mM Myriocin-SLNs stock solution was provided.

This solution was diluted in RPMI to achieve appropriate Myriocin concentrations. To study the SLNs permeation into the biofilm by confocal microscopy, we used SLNs labeled with 3,3-dioctadecyloxacarbocyanine perchlorate (DiO, Nanovector srl). A concentration of 100 mg/L was selected as a compromise to minimize nanoparticles aggregation but to allow their visualization at confocal microscope.

### In vitro antifungal susceptibility testing

The reference strain *Aspergillus fumigatus* Af293 and 20 clinical strains of *A. fumigatus* were used in this study. Clinical isolates were obtained from respiratory secretions of cystic fibrosis patients, during routine follow up. For the study, no additional data or samples were used. Therefore, neither ethical approval nor patient consensus was considered necessary.

Antifungal susceptibility testing was performed according the EUCAST protocol for planktonic cells [32]. We tested various concentrations of Myriocin, ranging from 0.25 to 64 mg/L. Readings were carried out after 48 h of incubation at 37 °C. The minimum effective concentration (MEC), the lowest drug concentration resulting in aberrant hyphal growth, was assessed.

In vitro susceptibility testing on biofilm-organized isolates was determined using a 96-well polystyrene plate, as described by Pierce et al [33]. Briefly, *A. fumigatus* strains were grown on Potato Dextrose agar (PDA) slants for 5 days at 30 °C. Conidia were harvested by adding 1.5 mL of PBS containing 0.025 % Tween-20 (v/v) and rocking gently. Conidia were then recovered and counted by hemocytometer. 10^6^ conidia/mL were grown in RPMI 1640 at 37 °C to induce biofilm formation. Biofilm-forming ability was quantified at 48 h by the 2,3-bis(2-methoxy-4-nitro-5-sulfophenyl)- 2H-tetrazolium-5-carboxanilide inner salt (XTT) reduction assay for metabolically active cells and crystal violet (CV) staining for total biomass measurement. To test the activity on biofilms, Myriocin, either alone or SLNs-loaded, was added at different concentrations (from 2 to 64 mg/L) to 24 h preformed biofilms and incubated for additional 24 h. The concentration of Myriocin that resulted in a 50 % reduction of biofilm metabolic activity was considered the sessile MIC. The experiments were performed in triplicate.

### Time-killing curve

Aliquots (10 mL) of the reference strain Af293 conidial suspension (1x10^5^–2x10^5^ conidia/mL) were added to 10 mL of RPMI medium alone (control) and to amphotericin B (concentration range 2–16 mg/L) voriconazole (1–8 mg/L) or Myriocin (from 2 to 16 mg/L) diluted in 10mL RPMI. Cultures were placed on the shaker and agitated at 37 °C. At predetermined time points (0, 2, 4, and 24h) two samples of 0.1mL were removed from each test suspension, serially diluted, and 0.1 mL aliquots were spread in duplicate on PDA plates and incubated at 37 °C for 48h. The time-kill curves were constructed by plotting the colony forming units (CFU) per milliliter (expressed as log10 CFU/mL) surviving at each time point in the presence of various antimicrobial agents.

### Confocal laser scanner microscopy (CLSM)

In order to understand the possible mechanism of the direct antifungal activity, we performed microscopic analysis on the reference strain Af293, isolated from the respiratory tract of a cystic fibrosis patient.

Fungal biofilms were formed on 18-mm-diameter round coverslips (Sarstedt, Italy) seeded in a 24-well plate. After incubation at 37 °C for 48 h, Myriocin 4 mg/L was added and the biofilm incubated for further 24 h. The coverslips were gently washed with PBS, stained with Calcofluor White (0.05 % v/v; Sigma), and mounted on a glass coverslip for confocal laser-scanning microscope (Leica, Inc.) visualization. Serial sections in the x-y plane were obtained along the z-axis. Three-dimensional reconstructions of imaged biofilms were obtained using associated software.

To determine whether Myriocin contained in SLNs penetrates the fungal biofilm, 1 μL SLNs labeled with DiO was added to a 48h-preformed biofilm and examined by CLSM, after 2 and 6 h, for the presence and localization of the fluorophore.

### Transmission electron microscopy (TEM)

For ultrastructural imaging, fungal biofilms were grown and treated as previously described for CLSM. Biofilms were fixed with 2.5 % glutaraldehyde in RPMI culture medium for 4 h at room temperature, rinsed in the same medium and then in double distilled water. Post-fixation was carried out in 1 % OsO_4_ in H_2_O for 2 h, in order to maintain also the lipid component of the membranes. The specimens were embedded in 2 % agar in H_2_O and then were dehydrated and embedded in epoxy resin. Ultrathin section were stained with uranyl, and observed in a Zeiss EM900 electron microscope operated at 80kV equipped with a 30 μm objective aperture.

### LC-MS analysis

Fungal cells were grown in 6-well plate, treated with Myriocin 4 mg/L for 24–48h, scraped and collected. Total lipids were extracted from both planktonic and biofilm-organized fungal cells by a two steps extraction. Samples were extracted with chloroform/methanol (17:1, v/v) for 120 min. The lower organic 17:1 phase lipid extract was collected. The remaining aqueous sample material was re-extracted with chloroform/methanol (2:1, v/v) for 120 min. The lower organic 2:1 phase lipid extract was collected. The lipid extracts, collected from both extractions, were vacuum evaporated [34, 35]. Finally, the lipid extracts were dissolved in 100μL chloroform/methanol (1:2, v/v) fortified with the internal standard, prepared and analyzed as previously described by Munoz-Olaya et al. [36]. Total phospholipids were quantitated and phytoceramide concentration was normalized onto total Pi-lipids content of each sample as previously described [37].

### Statistical analysis

All results represent the mean of at least three independent experiments. Comparative results were statistically analyzed using independent samples t-test (assuming equal variance) and ANOVA with GraphPad software. A *p* value of 0.05 was considered to be statistically significant.

## Abbreviations

CF: Cystic fibrosis
SLP: Sphingolipids
GLS: Glycoshingolipids
SPT: Serine palmitoyl transferase
SLN: Solid lipid nanoparticles
CLSM: Confocal laser scanner microscopy
TEM: Transmission electron microscopy
MIC: Minimum inhibitory concentration
MEC: Minimum effective concentration
RPMI: Roswell park memorial institute
PDA: Potato dextrose agar
PBS: Phosphate buffered saline
CV: Crystal violet
XTT: 2,3-bis(2-methoxy-4-nitro-5-sulfophenyl) 2H-tetrazolium-5-caboxanilide
Dio: 3,3-dioctadecyloxacarbocyanine perchlorate
OM: Original magnification
SD: Standard deviation
